# Supplementary material for: A new automated chilled adult release system for the aerial distribution of sterile male tsetse flies
Source: PLoS One. 2020 Sep 28;15(9):e0232306. doi: 10.1371/journal.pone.0232306 (PMC7521752; doi:10.1371/journal.pone.0232306)
Supplement: S1 Fig — (PDF) [file pone.0232306.s001.pdf]

BSi

By aewo

AUTOMATED CHILLED RELEASE SYSTEM

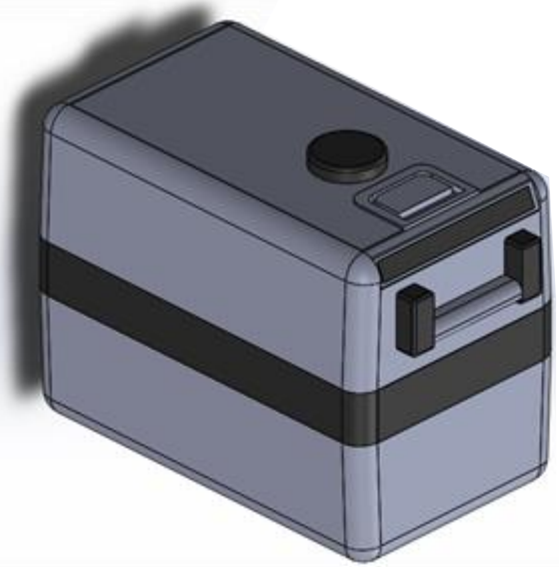

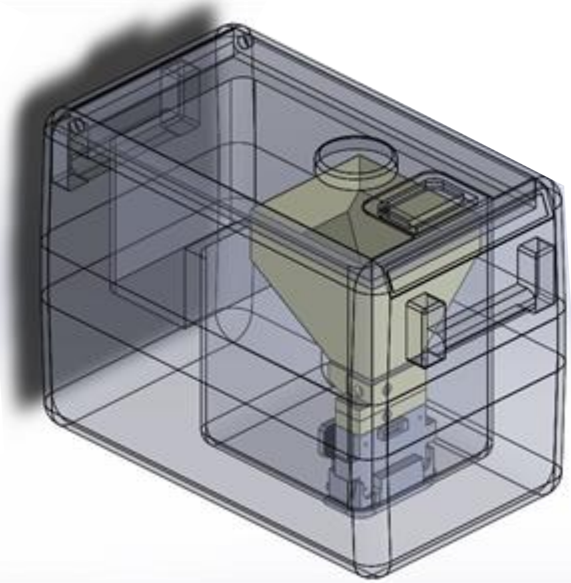

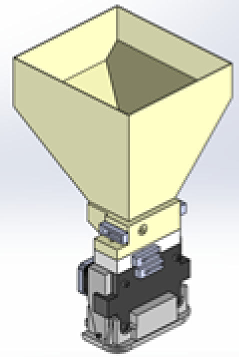

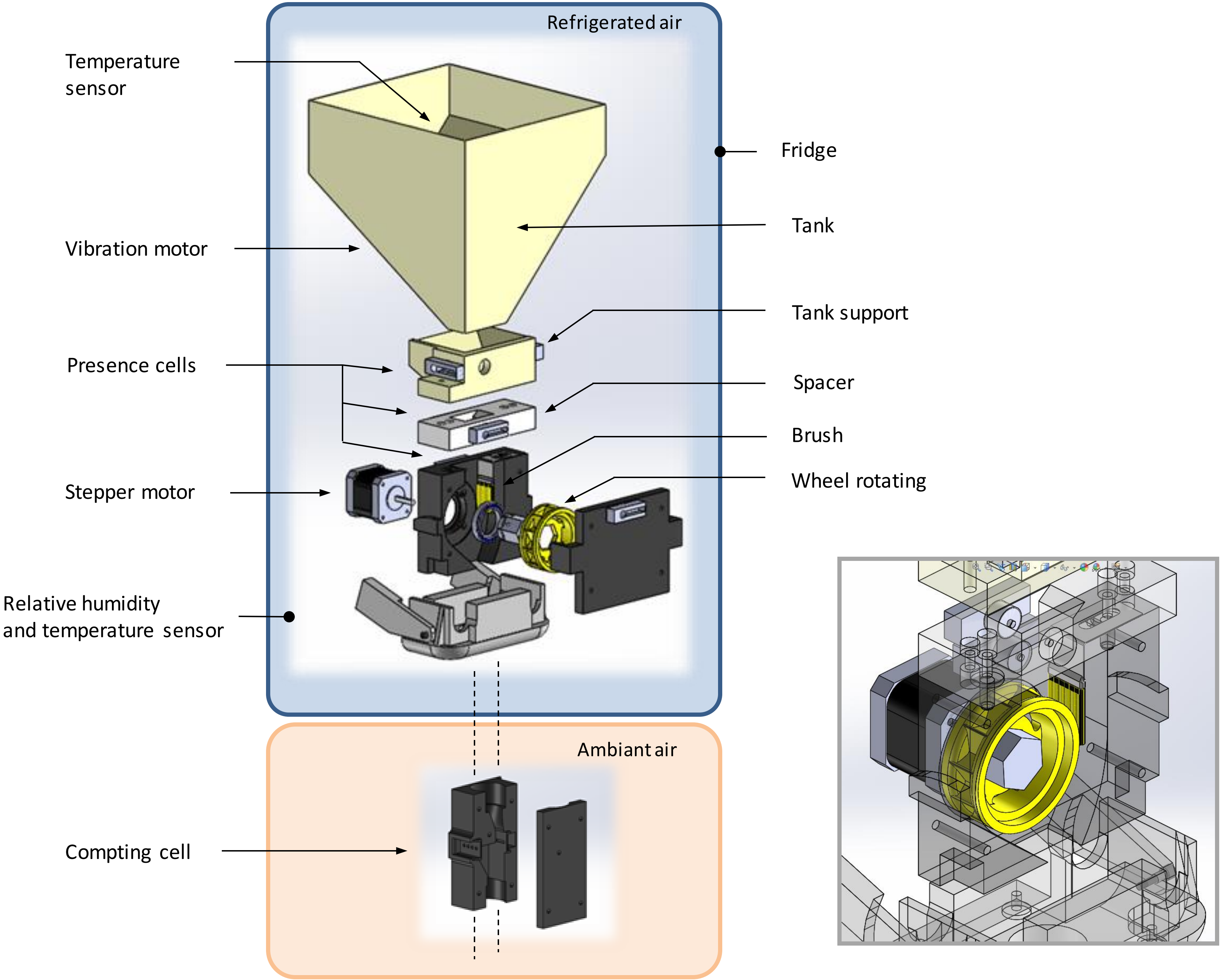
Refrigerated air
Temperature sensor
Fridge
Tank
Vibration motor
Tank support
Presence cells
Spacer
Brush
Stepper motor
Wheel rotating
Relative humidity and temperature sensor
Ambiant air
Compting cell

BSI Navigator

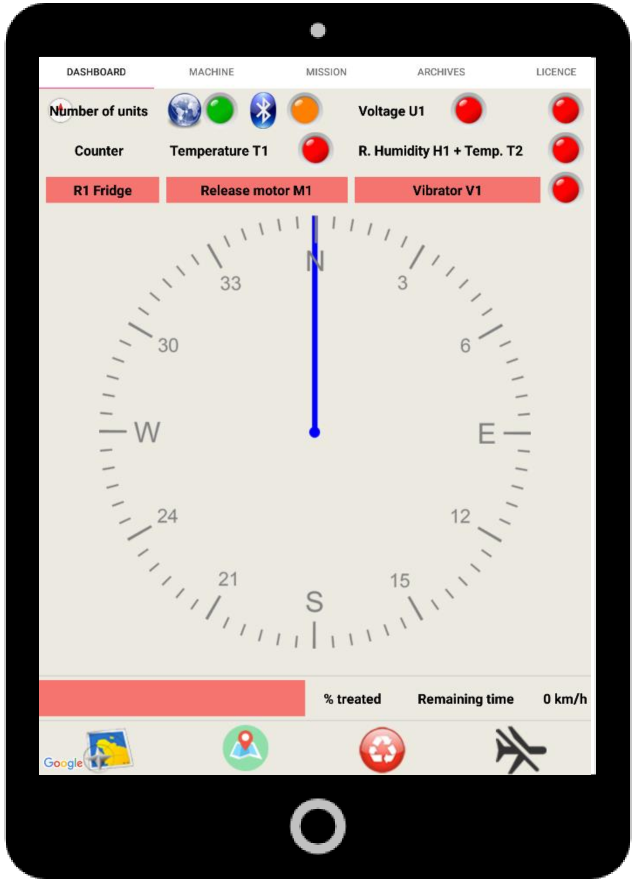

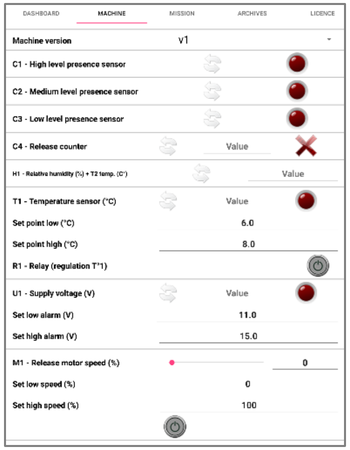

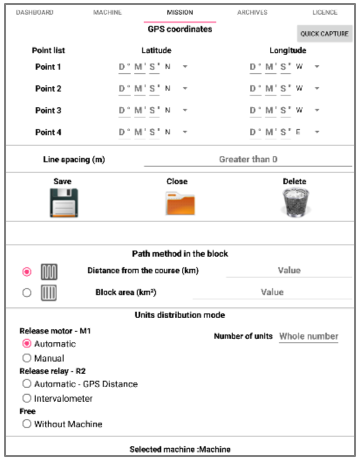

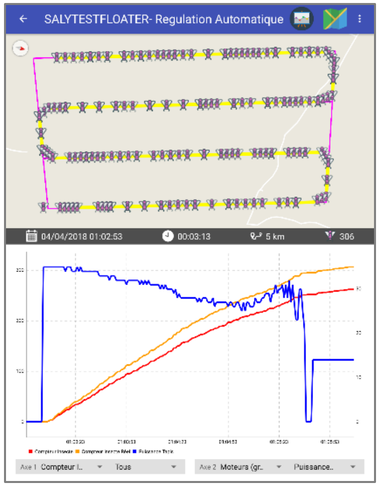

- Navigation dashbord
- Machine setting and calibration
- Mission management
- Report and sharing archives

BSI Controller

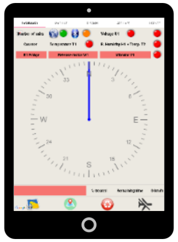

Command
- Stepper motor - DC motor - Relay
- Vibrator DC motor
- Fridge relay

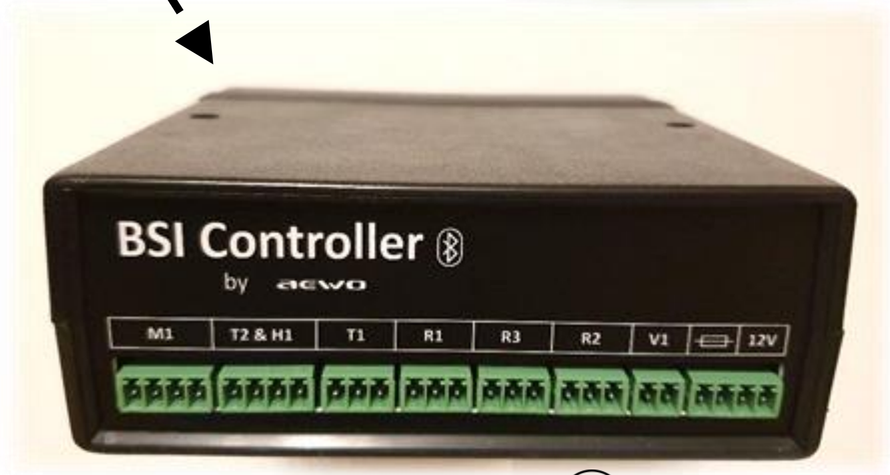

12 V power supply

Supervision
- Presence cells
- Counting cell
- Temperatures
- Relative humidiy
- Supply voltage

Auto rescue mode
